# VirDetect-AI: a residual and convolutional neural network–based metagenomic tool for eukaryotic viral protein identification

**DOI:** 10.1093/bib/bbaf001

**Published:** 2025-01-14

**Authors:** Alida Zárate, Lorena Díaz-González, Blanca Taboada

**Affiliations:** Doctorado en Ciencias, Instituto de Investigación en Ciencias Básicas Aplicadas (IICBA), Universidad Autónoma del Estado de Morelos, Cuernavaca, Morelos 62210, México; Centro de Investigación en Ciencias, Universidad Autónoma del Estado de Morelos, Cuernavaca, Morelos 62210, México; Departamento de Genética del Desarrollo y Fisiología Molecular, Instituto de Biotecnología, Universidad Nacional Autónoma de México, Cuernavaca, Morelos 62210, México

**Keywords:** viral metagenomic, eukaryotic virus identification, deep learning, convolutional neural networks, residual neural networks, VirDetect-AI

## Abstract

This study addresses the challenging task of identifying viruses within metagenomic data, which encompasses a broad array of biological samples, including animal reservoirs, environmental sources, and the human body. Traditional methods for virus identification often face limitations due to the diversity and rapid evolution of viral genomes. In response, recent efforts have focused on leveraging artificial intelligence (AI) techniques to enhance accuracy and efficiency in virus detection. However, existing AI-based approaches are primarily binary classifiers, lacking specificity in identifying viral types and reliant on nucleotide sequences. To address these limitations, VirDetect-AI, a novel tool specifically designed for the identification of eukaryotic viruses within metagenomic datasets, is introduced. The VirDetect-AI model employs a combination of convolutional neural networks and residual neural networks to effectively extract hierarchical features and detailed patterns from complex amino acid genomic data. The results demonstrated that the model has outstanding results in all metrics, with a sensitivity of 0.97, a precision of 0.98, and an F1-score of 0.98. VirDetect-AI improves our comprehension of viral ecology and can accurately classify metagenomic sequences into 980 viral protein classes, hence enabling the identification of new viruses. These classes encompass an extensive array of viral genera and families, as well as protein functions and hosts.

## Introduction

In recent decades, metagenomics has significantly improved our understanding of microbial life and viral diversity in diverse ecosystems [1], encompassing animals, plants, environmental samples, and the human body, among others. The persistent threat of infectious diseases, epitomized by the COVID-19 pandemic, highlights the pivotal role of metagenomics in pathogen surveillance and the need for early viral discovery to improve public health. However, the use of metagenomic data to detect and to characterize viruses is challenging due to the complexity of microbial communities and the lack of reference genomes. Moreover, the identification of novel viruses becomes an even greater obstacle due to the potential divergence in their genetic sequences from established viral genomes. Traditional methods such as sequence alignment and similarity searches are inadequate for capturing the breadth and intricacy of viral genomes.

Therefore, recent efforts have focused on developing tools that utilize artificial intelligence (AI) techniques to identify viral sequences. One of these tools is VirFinder [2], a binary method that detects prokaryotic viral nucleotide sequences by analyzing *k*-mer frequency. This method is based on the idea that phages and their hosts exhibit different *k*-mer signatures. Additionally, Bzhalava [3] developed an artificial intelligence (AI)-based binary classifier that discerns between viral and nonviral (NV) sequences. This method evaluated virus-related synonymous codon usage. Alshayeji [4] proposes another binary AI method for identifying viral contigs in human samples. This method utilizes *k*-mer counting and bag-of-words. Furthermore, Guo [5] proposes VirSorter2, a tool designed for the identification of DNA and RNA viruses, which utilizes a collection of customized automatic classifiers for specific viral groups to enhance the accuracy of virus sequence detection.

Moreover, deep learning approaches have also been used to identify viral genomes in metagenomic data. One example is ViraMiner, a binary classifier model that uses nucleotide data to distinguish viral and NV sequences in human samples [6]. Another is DeepVirFinder, a binary classifier model based on convolutional neural networks (CNNs) that distinguishes prokaryotic viruses from other entities [7]. EdeepVPP and hybrid EdeepVPP are CNN-LSTM (CNN–long short-term memory network) binary methods [8] developed to find new viruses by categorizing contigs as viral or NV. DLmeta is a CNN model that classifies metagenomic sequences as bacterial, viral, or plasmids using transformers [9]. Gene and protein domain identification underpin this approach. Virtifier is an attention-LSTM binary network that distinguishes between prokaryotic and nonprokaryotic viral sequences [10]. Finally, Virsearcher is an embedding-CNN binary model that uses gene information to label metagenomic contigs as either phages or nonphages [11].

The preceding approaches detect viral sequences in metagenomics samples efficiently. However, these methods exhibit limitations. Firstly, they are binary classifiers that distinguish between viral and NV sequences without providing details about the specific type of viruses identified. Second, these methods use nucleotide sequences, which are only used to identify viruses with high similarity to known sequences. Third, their performance relies on query sequence size, with better accuracy achieved for sequences over 500 bases. Furthermore, these techniques are often tailored to specific types of samples, such as samples from humans [3, 4, 6, 8, 9] or habitats with a high concentration of phages [2, 7, 10, 11], which limits their applicability in researching a broader spectrum of viral diversity.

In this work, we introduce VirDetect-AI, a novel tool for identifying eukaryotic viruses in metagenomic data from diverse habitats. This tool can identify both known and novel viruses with distant homologies. VirDetect-AI employs residual neural networks (ResNets) and CNNs to extract hierarchical and discriminative features from complex data. ResNets mitigate the problem of gradient loss through their residual connections, allowing deeper model training. Meanwhile, CNNs use local receptive fields to explore genomic data and spatial correlations, thereby facilitating the identification of sequence patterns. Therefore, VirDetect-AI improves classification accuracy and efficiency for both known and novel eukaryotic viral sequences. As a result, VirDetect-AI can reliably classify metagenomic sequences into 980 protein classes that cover 86 viral families and numerous genera. Moreover, this tool can not only contribute to a better understanding of viruses and their ecological impact but also has the potential to identify emerging viral pathogens, thus enabling more effective public health surveillance and response.

## Materials and methods

### Viral protein benchmark data

The virus protein database of the national center for biotechnology information (NCBI) (https://www.ncbi.nlm.nih.gov/labs/virus/vssi/#/) [12] was downloaded on 14 August 2021, filtering sequences with lengths between 100 and 14 000 amino acids. This upper limit was chosen to include viral proteins up to 13 500 amino acids long, as reported by Saberi [13]. In total, 9 930 462 sequences were obtained, along with their descriptions and family taxonomic level [14]. Host information was determined using the ViralZone platform [15].

Biological sequence databases often contain redundant sequences due to varying levels of research effort dedicated to different viruses. By removing nearly identical sequences, the over-representation of certain viral sequences can be mitigated, preventing classification models from becoming biased toward more frequent classes and ensuring a more balanced representation of different viral proteins. For this purpose, the CD-HIT tool v.4.8.1 (cd-hit-dup module) was used to group proteins with 98% identity [16, 17]. From each group, only the representative sequence was selected, resulting in a 63% reduction and yielding 3 695 997 unique viral amino acid sequences. Then, sequences exclusively infecting eukaryotic cells were isolated using host information (humans, vertebrates, invertebrates, plants, and fungi). This process culminated in a final set of 2 278 942 protein sequences belonging to 113 eukaryotic viral families.

### Class definitions and labeling protocol

The nonredundant viral protein sequences were organized into homologous groups to establish classification classes. First, the 2 278 942 sequences underwent clustering at 80% identity and 90% coverage using the CD-HIT v.4.8.1 (cd-hit module). A clustering at 60% followed this. Then, the final clusters were generated using the psi-cd-hit module with a 30% identity cutoff. For slower but more accurate clustering, the -g parameter was set to 1. This hierarchical clustering approach (three consecutive clustering steps) reduces the number of sequences incorrectly grouped, addressing a limitation inherent in the greedy incremental clustering of the CD-HIT algorithm [17]. Moreover, the lower identity threshold contributes to capturing broader diversity within each class; it also identifies homologous fragments, reflecting both sequence diversity and evolutionary relationships. As mentioned, redundancy was removed for sequences with up to 98% similarity. Consequently, each of the 47 977 clusters produced had sequences that exhibited a similarity ranging from 30% to 97% with their respective representative sequences.

Results indicated that 44.3% (*n* = 21,446) of clusters contained a single sequence, accounting for only 1.2% of all sequences. Furthermore, 97.8% (*n* = 46,943) contained fewer than 31 sequences. Clusters over 30 sequences were retained, representing 2.2% (*n* = 1034) of total clusters and 47.7% of sequences. Four overrepresented groups with 95 346–434 710 sequences required reduction at 90%–97% identity. After manually regrouping 56 clusters, 978 eukaryotic viral classes were created. This step was necessary due to errors in the initial VirDetect-AI classification, where sequences were often misassigned to multiple groups, sometimes due to annotation errors in the representative sequences. To correct these errors, we first identified groups with frequent misassignments. Then, Blastp v.2.11.0 [18] (query coverage >40% and identity >30%) was used to compare these sequences against the sequences of the groups to which the network had assigned them. Based on these comparisons, sequences were reassigned to their correct groups. These final 978 classes contained 1 013 722 sequences, representing 44.4% of the initial set. Detailed information about each of these classes, including the number of sequences, the number of viral families they comprise, their functions, and their hosts, among other attributes, is provided in Table S1. These classes encompassed information from all viral genome types (Table S2). Significantly, 76.1% (*n* = 86) of viral families persisted (Table S3), with 88.1% of classes containing sequences from one family. The model incorporates sequences from 521 viral genera (Table S4), a variety of viral protein functions (Table S5), and multiple viral hosts. These findings underscore the critical importance of a comprehensive representation of viral diversity within our model, which is essential for accurately identifying viruses in metagenomic data across a wide range of studies.

Finally, two negative classes were defined: the prokaryotic-viral class and the NV class. These classes are essential for achieving more accurate and reliable classification of eukaryotic viral protein fragments by clearly defining and managing different sequence types, including viruses that infect bacteria and archaea, as well as NV sequences. The prokaryotic-viral class comprised 1 187 888 bacteriophage sequences (Viral Protein Benchmark Data section). Utilizing the same hierarchical clustering strategy as described above, 6464 representative sequences were obtained. The NV class had 7561 sequences, including 2769 archaeal, 1317 fungi, 1872 bacterial, and 1576 human sequences. These sequences were retrieved from the NCBI protein database, accessed on 1 March 2023. Analysis using Blastp v.2.11.0 [18] (query coverage >40% and identity >30%) revealed that only 0.07% of prokaryotic viral sequences showed similarity with the viral eukaryotic classes, with identity scores ranging from 30% to 60%. In contrast, 0.42% of NV sequences showed similarity with viral eukaryotic classes, with identity scores ranging from 30% to 86%.

### Deep learning model

VirDetect-AI is a deep learning model built on ResNet and CNNs, designed to identify eukaryotic viral protein sequences. VirDetect-AI classifies amino acid sequences from metagenomic contigs’ different viral protein classes including eukaryotic-viral (978 classes), prokaryotic-viral (1 class), and NV (1 class).

#### Fragmentation of sequences into *k*-mers

**Figure 1:**
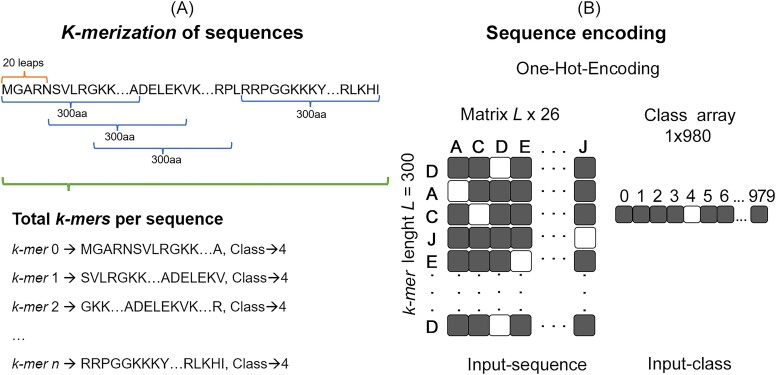
Encoding inputs for the VirDetect-AI model. (A) Fragmentation of sequences into 300-length *k*-mers with a 20-step leap. (B) Encoding of sequences using the one-hot encoding method, where amino acid *k*-mers are represented by their corresponding numeric labels.

The 1 027 747 amino acid sequences were fragmented into 300 amino acid *k*-mers with 20 leaps (Fig. 1A). *K*-mers containing up to 20% of “X” (undefined amino acid) were eliminated, resulting in 19 153 996 *k*-mers. Then, this dataset was partitioned into ~80% for training (15 342 350 *k*-mers), 10% for validation (1 896 246 *k*-mers), and 10% for testing (1 915 400 *k*-mers), using stratification by class to ensure that the same class proportions were maintained across all subsets. The training set was employed to fine-tune the model’s internal parameters, enabling it to recognize the patterns linked to each class. The validation set assisted in fine-tuning the model’s hyperparameters and evaluating its overall performance across iterations to avoid overfitting and underfitting. Finally, the test set offered an impartial assessment of the fully trained model, comprising data sequences previously unseen by the model that were not part of the training or validation processes.

#### Sequence encoding process

A preprocessing step was implemented to convert each amino acid within the *k*-mers into a numerical representation using one-hot encoding (Fig. 1B). The *k*-mers were then encoded into an *L* × 26 binary matrix, where *L* is the *k*-mer length (300 in this case), and 26 denotes the unique amino acid letters to be encoded. Each row in the binary matrix corresponds to the numerical representation of an amino acid. Similarly, the numeric labels for each of the 980 classes were encoded using one-hot encoding (Fig. 1B). Thus, the network’s inputs consist of the *k*-mers represented as *L* × 26 matrices and the class labels represented as a 1 × 980-dimensional array, where 980 denotes the total number of classes. The network’s output is a 980-dimensional array, with each value representing the probability of the *k*-mers belonging to 1 of the 980 classes.

#### Architecture of VirDetect-AI model

**Figure 2:**
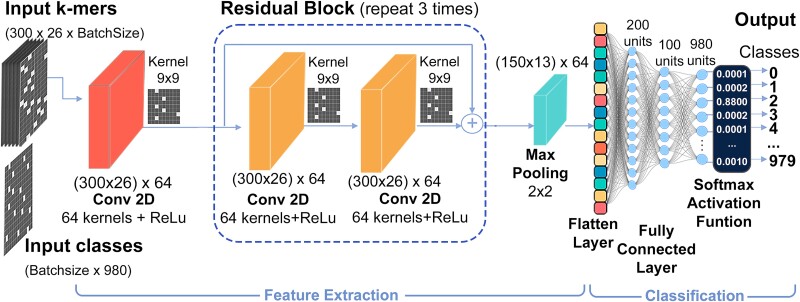
Architecture of the VirDetect-AI deep learning model, which integrates ResNet and CNN architectures.

Figure 2 depicts the architecture of the developed model, which consisted of a residual artificial neural network featuring convolutional layers within each ResNet-CNN residual block [19]. The first layer of the model was a convolutional layer (Conv2D), comprising 64 kernels with a kernel size of 9 × 9 and employing a Rectified Linear Unit (ReLU) activation function. This layer served to extract the primary feature maps.

Then, three residual ResNet-CNN blocks were added, each containing two Conv2D layers with properties identical to the initial Conv2D layer. Batch normalization layers were applied after each block to normalize the preceding layer activations. Skip connections were employed after each residual block to connect activations from the previous block with those of the current block, mitigating the gradient vanishing issue. Following the residual blocks, a Max-pooling layer with a pool size of 2 × 2 and strides of 2 was added, enhancing feature activations smoothness and computational efficiency.

The output of the Max-pooling layer was flattened and fed into a fully connected network consisting of two layers with 200 and 100 neuron units, respectively. The final dense layer with SoftMax activation generated a 980-sized array of class probabilities ranging from 0 to 1, where the highest probability determined the label assignment. This approach enhances user confidence by providing a probability value that indicates the certainty of the model in its classification. Table 1 presents the structure and parameters of the proposed deep learning model, implemented using Python 3 and Keras with TensorFlow [20, 21].

**Table 1:** Structure and number of parameters used at the different levels of the proposed model

**Step**	**Operations**	**Output dimension**
Input layer	One-hot encoding	300 × 26
Convolution layer	Conv2D (filter 64, kernel size: 9 × 9)	300 × 26
3x Residual Block	Batch normalization	300 × 26
	Activation (ReLU)	300 × 26
	Conv2D (filter 64, kernel size: 9 × 9)	300 × 26
	Batch normalization	300 × 26
	Activation (ReLU)	300 × 26
	Conv2 (filter 64, kernel size: 9 × 9)	300 × 26
Max-pooling layer	Pool size: 2 × 2, stride: 2	150 × 13
Flatten layer	Flatten	124 800
Fully connected layer	FC 200	24 960 200
Fully connected layer	FC 100	20 100
Output layer	SoftMax	980

#### Training, hyperparameter tuning, and testing procedures

At the end of each epoch, the model’s performance was evaluated by tracking both training and validation loss curves to assess its ability to generalize to unseen data. The optimal performance point was determined as the epoch where the validation loss stopped decreasing or began to diverge from the training loss, indicating the onset of overfitting. The final model selected for testing was the one that achieved the lowest validation loss with minimal divergence from the training loss. The model’s weights were adjusted at each epoch to minimize the categorical cross-entropy loss function [22]. The Adam optimizer [23] was utilized with a mini-batch size of 128 sequences per iteration. In total, 27 076 336 parameters were fine-tuned during training. Training was executed on a machine equipped with Intel(R) Core (TM) i9-12900K 12th Gen processors at 3.2 GHz and 128 GB of random access memory (RAM). To accelerate training, an NVIDIA GeForce RTX 3080 graphics processing unit (GPU) with 12 GB of memory was used. The training phase lasted 5 days and 13 h.

The search for the optimal model hyperparameters involved conducting a grid search using the parameters listed in Table S6A. The aim was to identify the configuration that maximizes accuracy while minimizing the loss function value. Model performance was tracked using the Weight & Biases web platform (https://www.wandb.com/) [24], enabling precise modeling to determine the optimal combination of hyperparameters.

In addition to hyperparameter tuning, selected ablation experiments were conducted to assess the impact of modifying key components of the model architecture. The variations tested included changes in the number of residual blocks, the structure of the CNN (number of convolutional layers, filter sizes and number of filters), the number of fully connected layers, and the use of the NV class, as detailed in Table S6B.

#### Establishing thresholds for reliable predictions

**Figure 3:**
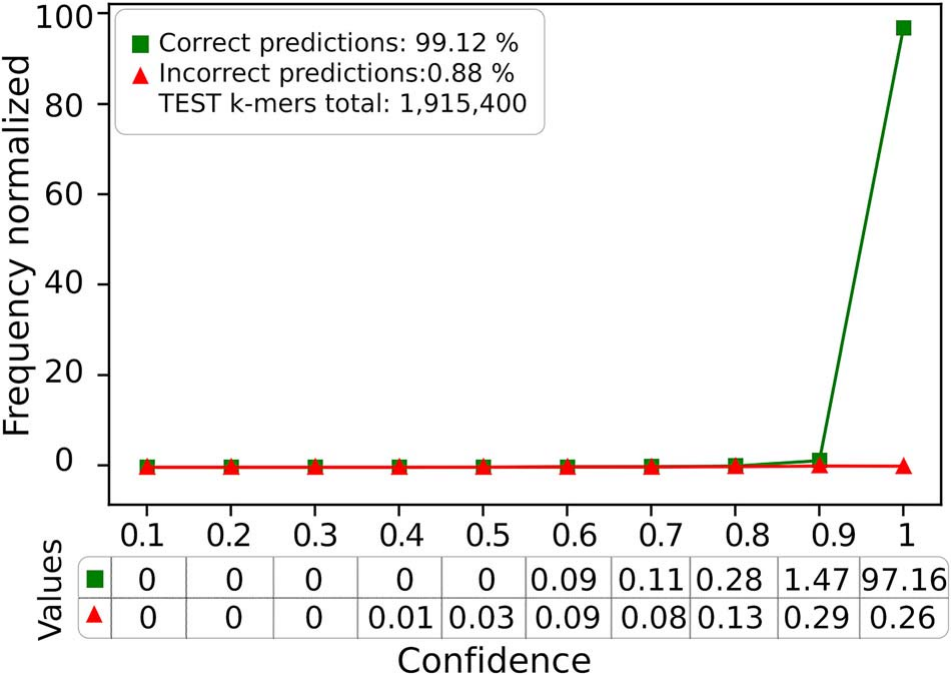
Confidence values for correct and incorrect predictions from the Test dataset.

For each *k*-mer, VirDetect-AI generates a probability value for belonging to each of the 980 classes, selecting the class with the highest probability. However, in certain instances, the highest probability value may be relatively low (e.g. 0.1, 0.2, 0.3, 0.4), indicating potential inaccuracies in the prediction and possible misclassification. Hence, establishing a minimum confidence value for reliable *k*-mer classification was crucial. To establish this threshold, the Test dataset was evaluated, and the correctly and incorrectly classified *k*-mers were plotted (Fig. 3). Remarkably, 98.31% of correctly predicted *k*-mers had confidence values >0.80, with only 0.81% falling below this threshold. In contrast, for incorrectly predicted *k*-mers, only 0.49% of the confidence values exceeded this value. These findings affirm the reliability of the model’s predictions when confidence values exceed 0.80.

#### Prediction process overview

The process of determining predictions for each query sequence involves several steps. Initially, input sequences are *k*-merized. Each resulting *k*-mer is predicted by VirDetect-AI, yielding a class and confidence value. Each sequence consists of *n k*-mers and their corresponding class predictions. *K*-mers with a confidence score below 0.80 are removed. If all *k*-mers are discarded, the sequence is classified as “Unknown.” Afterward, the remaining *k*-mers are categorized according to their projected classes and arranged in descending order based on the total count of *k*-mers in each class. The prediction for the sequence is determined by selecting the class with the highest number of assigned *k*-mers. In the case of a tie, where two or more classes have the same number of *k*-mers, the class with the highest average confidence value among its *k*-mers is selected as the prediction. A query coverage percentage is computed to evaluate the confidence of the prediction for each sequence. This percentage represents the proportion of *k*-mers that contribute to the final prediction compared to the total *k*-mers per sequence, including those that fall below the confidence threshold.

The output of VirDetect-AI includes a prediction final report file, a *k*-mer report file, a sequence of unknown report files and six graphs, which collectively provide a comprehensive analysis of the sequence data. Unlike other classifiers that may only distinguish between viral and NV sequences, VirDetect-AI offers detailed insights by classifying sequences into 980 groups of eukaryotic viral proteins. The graphs depict total sequence classification, viral sequence classification, predicted viral families and genera, protein functions, and viral hosts. The report also provides a query coverage value, which is essential for result analysis. This value indicates the percentage of the query sequence that the prediction has covered. A higher coverage and confidence value indicate a more precise classification of known viruses, while lower coverage and higher confidence could suggest a novel virus. This is attributed to VirDetect-AI’s enhanced precision in identifying conserved motifs within protein groups for novel viruses.

### Evaluation metrics

The cross-entropy loss function was used to measure the error between the predicted and true outputs [25]. VirDetect-AI evaluation metrics included accuracy [equation (1)], precision [equation (2)], sensitivity (true positive rate) [equation (3)], specificity [equation (4)], and false positive rate [equation (5)]. Additionally, F1-score [equation (6)] and Matthew’s correlation coefficient (MCC) [equation (7)] addressed the sensitivity–precision trade-off, crucial for imbalanced class problems.


(1) \begin{equation*} Accuracy=\frac{TP+ TN}{TP+ TN+ FP+ FN} \end{equation*}

(2) \begin{equation*} Precision=\frac{TP\ }{TP+ FP\ } \end{equation*}

(3) \begin{equation*} Sensitivity\ or\ TPR=\frac{TP}{TP+ FN} \end{equation*}

(4) \begin{equation*} Specificity=\frac{TN}{TN+ FP}\kern0.75em \end{equation*}

(5) \begin{equation*} FPR=1- Specificity \end{equation*}

(6) \begin{equation*} F1- score=\frac{TP}{TP+\frac{1}{2}\ \left( FP+ FN\right)} \end{equation*}

(7) \begin{equation*} MCC=\frac{TP\times TN- FP\times FN}{\sqrt{\left( TP+ FP\right)\left( TP+ FN\right)\left( TN+ FP\right)\left( TN+ FN\right)}} \end{equation*}

Additionally, precision, sensitivity, and F1-score metrics were computed for each class individually. True positive (TP) indicates correctly classified *k*-mers belonging to the evaluated class; true negative (TN) denotes correctly classified *k*-mers not belonging to the evaluated class; false positive (FP) represents incorrectly classified *k*-mers not belonging to the evaluated class but incorrectly classified as such; false negative (FN) signifies *k*-mers belonging to the evaluated class but incorrectly classified into other classes.

Finally, to comprehensively assess model performance, global metrics like accuracy, sensitivity, F1-score, and MCC were calculated by averaging the values across all classes.

### Comparative and performance analysis

#### New viral-NCBI sequences

To evaluate the performance of VirDetect-AI and its novel classification approach, we conducted a comparative analysis against two widely adopted viral metagenomic classifiers: DeepVirFinder [7] and VirSorter2 [5]. Unlike VirDetect-AI, which operates at the amino acid level, DeepVirFinder and VirSorter2 analyze viral sequences at the nucleotide level. DeepVirFinder employs a binary classification system, distinguishing sequences as either viral or NV. For this analysis, default settings were applied, and sequences with a *P*-value <.001 were considered viral. In contrast, VirSorter2 offers a more nuanced classification, sorting sequences into five distinct viral categories: double-stranded DNA (dsDNA) phages, nucleocytoplasmic large DNA viruses (NCLDVs), RNA viruses, single-stranded DNA (ssDNA) viruses, and *Lavidaviridae*. This tool was also run with default parameters.

For the comparative analysis, nucleotide viral sequences deposited in the NCBI Virus database after the creation of VirDetect-AI were downloaded. These sequences included newly identified viral species as well as previously known species with newly annotated sequences. In total, 434 883 sequences were obtained (accessed on 19 January 2024), covering the period from 1 September 2021, to 31 December 2023, excluding sequences shorter than 900 nucleotide length and SARS-CoV-2 sequences. Additionally, SARS-CoV-2 sequences were obtained, with up to 20 sequences randomly selected and stratified by country, in total 1737 sequences were obtained. Metadata, including family, genus, species, molecule type, host, and GenBank title, was used to differentiate between eukaryotic (*n* = 425 847) and prokaryotic (*n* = 9036) viral sequences. SeqKit v.2.5.1 [26] was used to subsample 1% of eukaryotic viral sequences (*n* = 4223), 3% of SARS-CoV-2 sequences (*n* = 45), and 4% of caudoviricetes viral sequences (n = 420). The final NCBI-EukVirusN-set comprises 4268 nucleotide sequences (Table 2), which are the subsample eukaryotic viral sequences and SARS-CoV-2, with a minimum sequence length of 300 bp, a maximum sequence length of 235 329 bp, and a median length of 2151 bp (interquartile range (IQR) [1410-2913]). The NCBI-CaudoN-set comprises 420 nucleotide sequences (Table 2), with minimum sequence length of 957 bp, maximum sequence length of 378 590 bp and median length of 42 277 bp (IQR [23 851–56 811]).

**Table 2:** Information of datasets evaluated.

**Dataset**	**Description**			**Statistics on the length of sequences**			
	**Type**	**# Seqs**	**Origin**	**Min**	**Max**	**Median**	**IQR**
NCBI-EukVirusN-set	Nucleotides	4268	Virus NCBI database	900	235 329	2151	1410–2913
NCBI-CaudoN-set	Nucleotides	420	Virus NCBI database	957	378 590	42 277	23 851–56 811
NCBI-EukVirusP-set	Amino acids	36 660	Virus NCBI database	300	8927	574	409–833
Meta-EukVirus-set	Amino acids	703	Metagenomic from 120 Oropharyngeal samples	300	4557	606	419–2595
Meta-Unknown-set	Amino acids	113	Metagenomic from 120 Oropharyngeal samples	300	516	332	312–365
Meta-Human-set	Amino acids	1280	Metagenomic from two Oropharyngeal samples	300	1720	369	326–446
Meta-Bacteria-set	Amino acids	2428	Metagenomic from a single infant fecal sample	300	3172	434	354–545

To use these two datasets with VirDetect-AI, open reading frames (ORFs) were predicted for both the NCBI-EukVirusN-set and the NCBI-CaudoN-set using Prodigal v2.6.3 [27]. In total, 10 736 ORFs longer than 300 amino acids (aa) were identified in the NCBI-EukVirusN-set, and 6302 ORFs longer than 300 aa were detected in the NCBI-CaudoN-set.

To further assess the performance of VirDetect-AI on new eukaryotic viral protein sequences from NCBI, which may exhibit characteristics distinct from their nucleotide counterparts, additional amino acid sequences were downloaded. The same filtering criteria, including date range and sequence length, as described above, were applied to ensure consistency across all datasets. These sequences, not included in VirDetect-AI’s development, comprised 3 205 729 viral proteins and 1840 SARS-CoV-2 proteins. After excluding sequences shorter than 300 amino acids, 1 130 538 sequences remained for analysis. Clustering with CD-HIT (version 4.8.1) at 80% similarity reduced redundancy, resulting in 94 825 representative sequences. Metadata was used to filter eukaryotic viral proteins, resulting in a final dataset of 36 660 sequences, designated as the NCBI-EukVirusP-set (Table 2), with a median length of 574 amino acids (IQR [409–833]). This dataset will not be used for comparison with DeepVirFinder and VirSorter2, which operate at the nucleotide level. However, evaluating VirDetect-AI on it is crucial for testing its ability to generalize novel viral proteins and for assessing its robustness.

#### Real metagenomic data

These datasets play a crucial role in ensuring the robustness of VirDetect-AI against real-word, noise, and contamination data. Moreover, they offer valuable insights into the practical applications of the model, such as early viral detection and surveillance. By closely resembling the richness and diversity of actual biological samples, these sets enhance the model’s ability to perform effectively in real-world scenarios. Prior to analysis, preprocessing steps were applied to all metagenomics data. Briefly, Fastp v.0.20.0 [28] was used to trim adapters and low-quality bases from the 5′ and 3′ ends, as well as reads with low complexity or <40 bases. Exact duplicate reads were removed with CD-HIT-DUP v.4.8.1 [29]. Bowtie 2 v2.3.4.3 [30] was used to remove human host and ribosomal reads using Silva database v.132 and the human genome (GRCh38.p13), respectively. The remaining readings were considered valid and were used for further analysis.

A comprehensive analysis was conducted on 120 metagenomics datasets obtained from human oropharyngeal specimens [31], focusing on COVID-19-positive patients across varying illness severity levels, from outpatient cases to hospitalizations and fatalities (Table 2). Initially, 200 million reads were assembled using Spades v.3.13.0 [32], resulting in 16 297 contigs ≥900 nucleotides. CD-HIT clustered these contigs at 95% identity to derive unique sequences. Subsequent ORF prediction with Prodigal v2.6.3 yielded 816 sequences, each ≥300 amino acids (aa). Blastp v.2.11.0 (query coverage >40% and identity >30%) analysis against the NCBI nonredundant (NR) protein database identified 703 ORFs with homology to eukaryotic viruses (30%–100% identity), a median length of 606 aa (IQR [419–2595]). Additionally, 113 ORFs with no known homologous proteins were detected, with a median length of 332 aa (IQR [312–365]). These sequences comprised the Meta-EukVirus-set and Meta-Unknown-set (Tables 2), respectively. It’s important to note that the NR database utilized aligns with those referenced in previous publications.

To evaluate VirDetect-AI’s performance with NV sequences, two negative datasets were utilized: one comprising human sequences and the other bacterial sequences. For the Meta-Human-set (Table 2), human-derived reads from samples 188 and 207, selected from the original set of 120 samples [31], were used. A total of 59 224 264 reads were assembled into 24 516 contigs. These contigs yielded 1280 ORFs, each ≥300 amino acids (aa), with a median length of 369 aa (IQR [326–446]). Regarding the bacterial dataset, sequences were extracted from fecal samples of two infant subjects [33]. This dataset comprises 4898 protein sequences from Bifidobacterium bacteria, the predominant species in the newborn gut [34]. Following the methodology described in Rivera *et al*. [33], contigs ≥300 aa were filtered, resulting in 2428 sequences constituting the Meta-Bacteria-set (Table 2), with a median length of 434 aa (IQR [354–545]).

These four metagenomics datasets were essential for comparing VirDetect-AI’s performance with Blastp. Unlike nucleotide-level classifiers, Blastp enables direct protein sequence comparisons, providing critical insights into the model’s ability to accurately identify and differentiate viral proteins from NV sequences. Additionally, Blastp’s capability to detect distant homologs offers a deeper evaluation of the model’s sensitivity, revealing relationships that nucleotide-based tools might overlook.

## Results and discussion

The subsequent subsections outline the training results of the deep learning model, assess its performance on the Test dataset, and evaluate its effectiveness on experimental metagenomic benchmark datasets.

### Evaluation of VirDetect-AI’s performance during the training process

**Figure 4:**
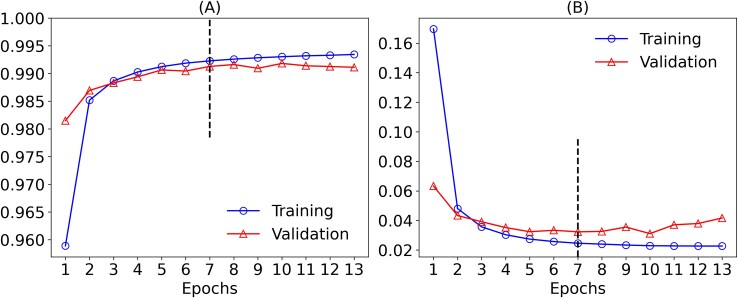
Training and validation performance of VirDetect-AI. (A) Accuracy evaluation. (B) Loss function analysis.

Figure 4 illustrates the training and validation performance of VirDetect-AI. By Epoch 7, the model achieved optimal performance without signs of overfitting. The blue line (circle symbol) represents the training process, with an accuracy of 0.99 (Fig. 4A) and a loss of 0.02 (Fig. 4B) in this epoch, highlighting the model’s robustness in classifying metagenomic sequences. The red line (triangle symbol) represents validation data, with an accuracy of 0.99 (Fig. 4A) and 0.03 loss (Fig. 4B), confirming its ability to generalize to unseen data. After this epoch, as shown by the dotted line in Fig. 4B, the discrepancy between validation and training losses began to increase, indicating a reduction in generalization capacity. To quantify this, the divergence in loss was calculated as the difference between the values of the training and validation sets at each epoch (Fig. S1). From Epoch 8, this divergence increased and started to fluctuate, suggesting a lack of convergence beyond this point, even reaching −0.0190 at Epoch 13. This finding supports the decision to select the model saved at Epoch 7, avoiding overfitting and preserving optimal real-world performance, despite training continuing until Epoch 13.

### Evaluation of VirDetect-AI’s performance on the Test dataset

**Figure 5:**
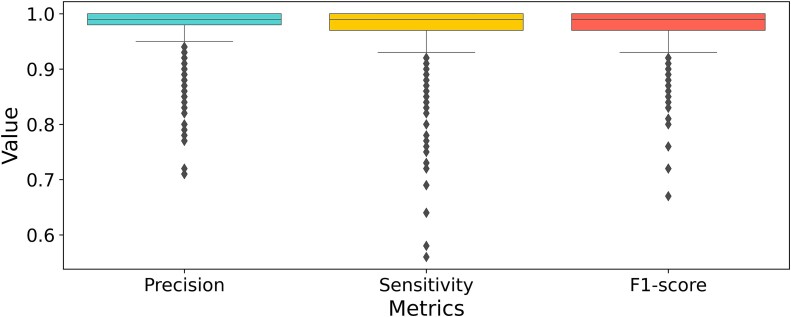
Performance of VirDetect-AI on the Test dataset. Boxplot showing the distribution of metrics (precision, sensitivity, and F1-score) for each of the 980 classes.

The performance evaluation of the trained model was conducted using the Test dataset, which comprised 1 915 400 *k*-mers previously unseen by the model. On Fig. 5, the distribution and variability of each metric across the 980 classes is illustrated. Notably, the results indicate outstanding performance across all metrics, with a sensitivity of 0.97, a precision of 0.98, and an F1-score of 0.98. Furthermore, the MCC, known for its robustness in assessing classification accuracy even in scenarios with unbalanced class distributions such as in this study, achieves a remarkable value of 0.99.

**Figure 6:**
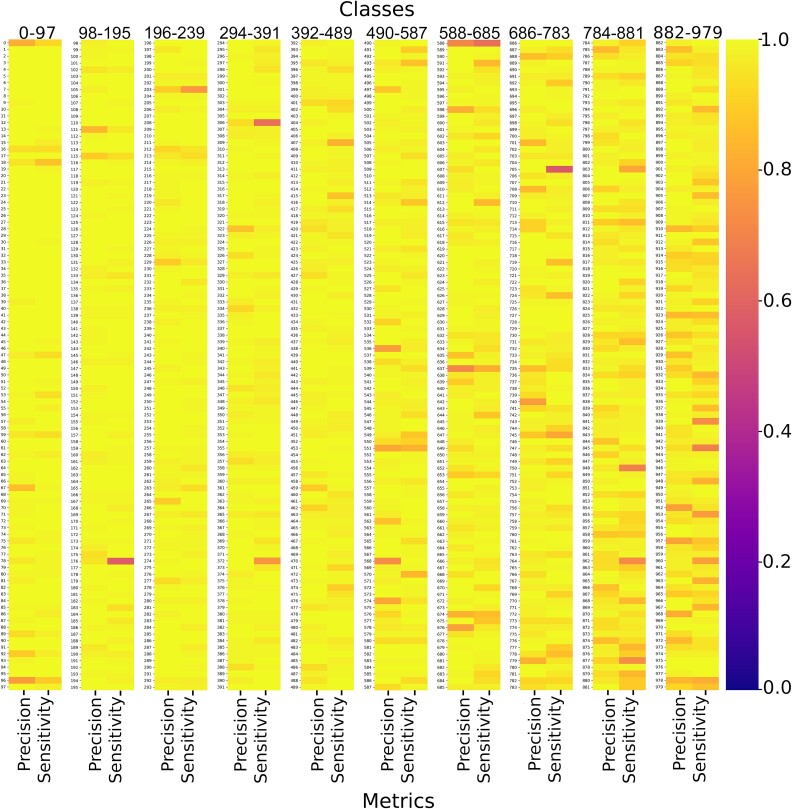
Precision and sensitivity metrics for each of the 980 classes on the Test dataset.

To provide a detailed evaluation of the model’s performance on the Test dataset, Fig. 6 presents a heatmap showing precision and sensitivity for each of the 980 classes. Overall, the values are consistently high across all classes, both in terms of classification within the 980 individual classes and in discriminating between viral and NV classes, underscoring the model’s robust performance. Notably, 94.4% of the classes exhibit a sensitivity above 0.9, while only 1.3% of the classes show sensitivity below 0.7. A comprehensive breakdown of these metrics, including the F1-score for each class, is provided in Table S7.

### Ablation study

**Figure 7:**
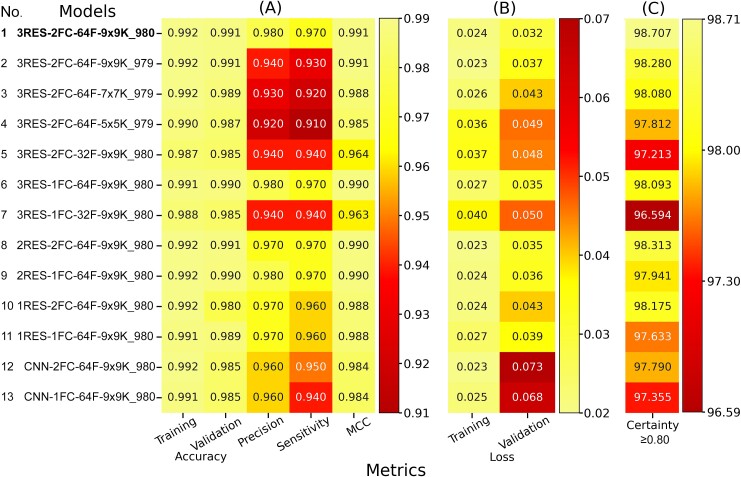
Heatmap results of the ablation study evaluating different models, divided into three sections: (A) training and validation accuracy, along with precision, sensitivity, and MCC obtained from the Test dataset; (B) training and validation loss; and (C) proportion of correctly predicted *k*-mers with certainty starting at a threshold of 0.80, referred to as the certainty threshold.

As described in the Materials and Methods section, an ablation study was conducted using selected examples to evaluate the impact of modifying key components of the VirDetect-AI model architecture (Model 1), as detailed in Table S6B. The results, presented in Fig. 7, demonstrate a performance degradation when these components were altered.

For instance, excluding NV class, Model 2, which otherwise shares the same architecture as Model 1, led to a notable decrease in performance, with precision dropping to 0.940 and sensitivity of 0.930. A reduction in filter size, as seen in Model 3 (7 × 7 filter) and Model 4 (5 × 5 filter), further decreased precision and sensitivity to 0.930/0.920 and 0.920/0.910, respectively, when compared to Model 2.

Additionally, reducing the number of filters from 64 to 32 in Model 5, compared with Model 1, caused both precision and sensitivity to drop to 0.940. Similarly, comparing Model 6 with Model 7 revealed a comparable decline in performance, with precision and sensitivity falling from 0.980/0.970 in Model 6 to 0.940/0.940 in Model 7. Reducing the number of residual (RES) blocks also had a pronounced effect; Model 8, with one fewer RES block than Model 1, showed a decrease in precision and sensitivity to 0.970, while Model 10, with two fewer RES blocks, exhibited even worse performance. Eliminating residual blocks entirely in Model 12 resulted in the most significant drop in both metrics.

Changes in fully connected (FC) layers had a smaller impact on performance compared to altering filters or RES blocks. While precision and sensitivity decreased slightly in models with fewer FC layers, these metrics remained high. For instance, Model 6, which is identical to Model 1 but with one fewer FC layer, maintained precision and sensitivity at 0.980 and 0.970, respectively. Similarly, Model 7 (compared to Model 5) and Model 10 (compared to Model 11) showed only minor reductions. The most notable effect of reducing FC layers was a decrease in certainty, which measures the model’s confidence in classifications (valid for values starting from 0.8). Models with fewer FC layers showed lower certainty, such as in Model 7 (96.594) compared to Model 5 (97.213), or Model 10 (98.175) compared to Model 11 (97.633), indicating that reducing FC layers slightly diminishes classification confidence.

These results confirm that the selected model (Model 1), provided the best overall performance across all metrics. Overall, the factors that most significantly influenced the model’s performance were: (i) the removal of the NV class, (ii) the reduction in the number of filters, and (iii) the elimination of residual blocks. By contrast, removing a single fully connected layer had the least impact, affecting primarily certainty while preserving precision and sensitivity.

### Evaluation on new viral sequences of NCBI database using VirDetect-AI, VirSorter2, and DeepVirFinder

The NCBI-EukVirusN set and NCBI-CaudoN set datasets (Comparative and Performance Analysis section) were analyzed using VirDetect-AI, VirSorter2, and DeepVirFinder. The primary objective was to evaluate the effectiveness of each tool in recovering viral sequences, which is the primary function of VirSorter2 and DeepVirFinder. Unlike VirDetect-AI, which classifies sequences into 980 classes and can reach the viral family level, VirSorter2 and DeepVirFinder are more limited in their classification depth. For VirDetect-AI, predicted ORFs at the amino acid level were used as input. A sequence was considered correctly classified if at least one predicted ORF matched the entire nucleotide sequence. Sensitivity (TP rate) was the primary metric used to compare the performance of the three tools, as both datasets consisted entirely of viral sequences.

The results show that VirDetect-AI demonstrated superior performance on the NCBI-EukVirusN-set, accurately recovering 87.7% of viral sequences (Table 3). In comparison, VirSorter2 and DeepVirFinder recovered only 32% and 16%, respectively. Notably, VirDetect-AI exhibited high sensitivity at the ORF level, classifying all predicted ORFs as viral, even when the full sequences were not complete. For the NCBI-CaudoN-set, VirSorter2 achieved a recovery rate of 95% for viral sequences, followed closely by VirDetect-AI with 93%, while DeepVirFinder recovered 57%. It is important to note that VirDetect-AI was not specifically optimized for prokaryotic viral sequence classification, as only 1 of its 980 classes was trained with such sequences. Furthermore, only a median of 64% (IQR [50%–81%]) of ORFs were predicted from the complete sequences. Despite these limitations, the tool demonstrated impressive performance, as detailed in Table 3.

**Table 3:** Comparison of our tool against the two most widely used tools for viral sequence detection. The datasets evaluated are recently uploaded viral sequences in the NCBI databases.

**Datasets**	**Tools comparison** **(Sensitivity)**		
	**VirDetect-AI** **(this work)**	**VirSorter2 v2.2.4**	**DeepVirFinder**
NCBI-EukVirusN-set (*n* = 4268)	0.87	0.32	0.16
NCBI-CaudoN-set (*n* = 420)	0.93	0.95	0.57

Moreover, the NCBI-EukVirusP-set protein sequences (Comparative and Performance Analysis section) were also analyzed using VirDetect-AI. The results indicated that 73.8% of the entities were classified as viral, 21.3% as NV, and 4.9% remained unclassified, thus categorized as unknown. Fig. S2 illustrates that among the viral entities (*n* = 27 068), 89.2% were classified into eukaryotic viral classes, while 10.8% fell into the prokaryotic viral class. Notably, sequences predicted to belong to eukaryotic viral classes (*n* = 24 132) were classified into 978 different eukaryotic classes. These classifications predominantly corresponded to families such as *Anelloviridae* (9.9%), *Mimiviridae* (8.1%), *Orthoherpesviridae* (6.6%), and *Poxviridae* (4.8%), among others. *Homo sapiens* emerged as the prevalent host, covering 36.6% of the results. Overall, VirDetect-AI successfully identified new sequences from 82 viral families, spanning 171 different hosts. It’s important to emphasize the similarity between the prevalent viral families predicted by the VirDetect-AI model and those identified in the original dataset.

NCBI-EukVirusP-set included 2154 protein sequences from 52 newly identified viral families, which were not part of the original data set of VirDetect-AI. Despite this, the model successfully identified 827 sequences (38.4%), as belonging to eukaryotic viruses, with these sequences spanning across 43 of the newly identified families (Fig. S3). This indicates that VirDetect-AI recognized at least one protein from 82.7% of the new families. Notably, the most prevalent family among the 43 detected was *Botourmiaviridae*, accounting for 28.9% (*n* = 239) of the sequences. This family includes viruses infecting plants and filamentous fungi [35]. Interestingly, many of these sequences were classified by ViDetect-AI as belonging to the *Tombusviridae* family (*n* = 293, 24.7%). This is consistent with existing literature that highlights the similarity between the *Ourmiavirus* genera within *Botourmiaviridae* family and members of the *Tombusviridae* family [36]. Furthermore, 56.5% of these sequences were classified into viral families predominantly associated with plants and/or fungal hosts, which aligns with the host range of the *Botourmiaviridae* family. The sequences that remained unclassified were confined to only 9 new eukaryotic families (Fig. S3), likely representing protein groups that were not adequately represented in the training data due to their low sequence abundance.

Regarding the NV entities representing false negative results of VirDetect-AI’s, the most prevalent families lost were *Orthoherpesviridae* (11%), *Nimaviridae* (9.4%), *Mimiviridae* (6.1%), *Narnaviridae* (5.9%), *Potyvirida*e (%), and *Retroviridae* (%), among others. These erroneous assignments could be attributed to several factors: (i) low-complexity sequences containing tandem repeats of amino acids are commonly associated with sequencing artifacts, contaminants, and/or conserved domains found in a broad spectrum of proteins from large bacterial, eukaryotic, and DNA viruses [37]. Examples of such conserved domains include ankyrin repeats or KilA-N domains [38]. (ii) contamination of publicly available databases, including viral sequence collections in GenBank and UniProt, which have been misidentified as hosts, vectors, and laboratory components [39]; (iii) high identity of false negative sequences with parts of some viral proteins; (iv) viruses integrated into the host genome, such as retroviruses, constituting 8% of the human genome [40]. Additionally, viruses like herpesviruses, adenoviruses, parvoviruses, polyomaviruses, arenaviruses, bornaviruses, filoviruses, and rhabdoviruses [41–43], among others, can integrate their genes into the host genome; and (iv) exclusion of classes with fewer than 30 sequences, which can impair VirDetect-AI’s ability to accurately identify rarer or less-represented viral proteins. Finally, despite the 4.9% of sequences that remained unclassified, the model successfully identified at least one representative protein from each of the 80 different families within this group of unclassified sequences.

### Evaluation of VirDetect-AI on real metagenomic datasets and comparison with Blastp

**Figure 8:**
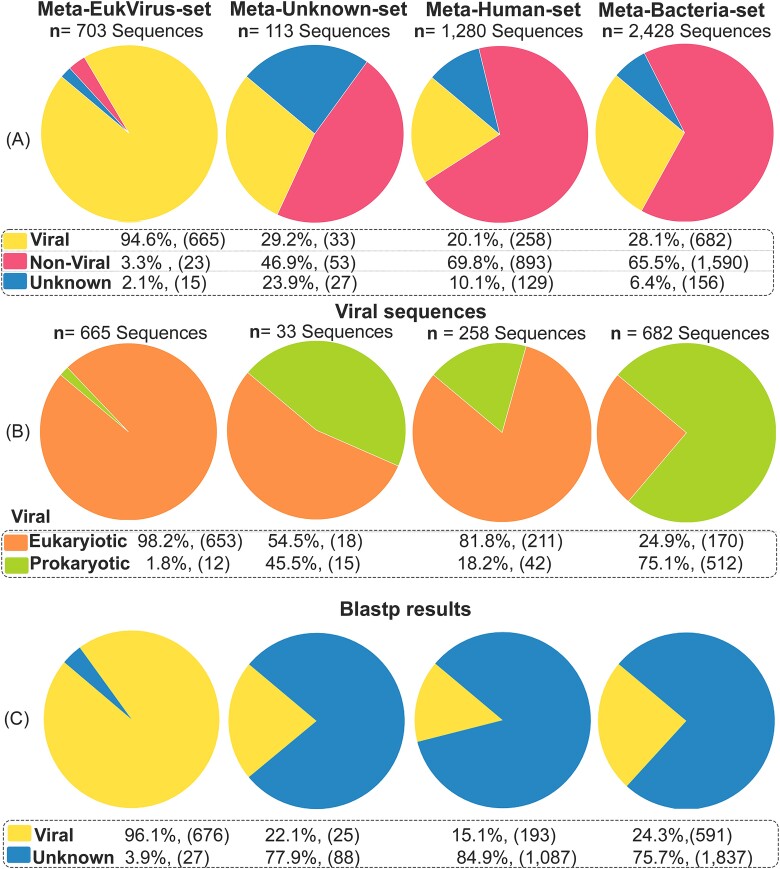
Classification results for real metagenomic data, including both positive and negative viral sequences. (A) Predicted classes by VirDetect-AI. (B) Viral types classified by VirDetect-AI. (C) Results from Blastp.

The VirDetect-AI model’s efficacy on real metagenomics datasets was assessed using the experimental metagenomic datasets described in Comparative and Performance Analysis section. The results from the Meta-EukVirus-set, which illustrate known and potential distant homologs of eukaryotic viruses, demonstrated that VirDetect-AI categorized 94.6% of the entities as viral, 3.3% as NV (archaeal, fungi, bacteria, or human origin), and 2.1% as unclassified or unknown (Fig. 8A). In Fig. 8B, it is shown that among the viral entities (*n* = 665), 98.2% were assigned to eukaryotic viral classes, while 1.8% were classified as prokaryotic-viral. Interestingly, 81% of the sequences were grouped into only eight classes (Table S8), with Class 1 (37.3%), Class 14 (15.2%), and Class 3 (15%) being the most prevalent. These findings suggest a relative lack of viral diversity within the datasets originating from COVID-positive patients [31].

**Figure 9:**
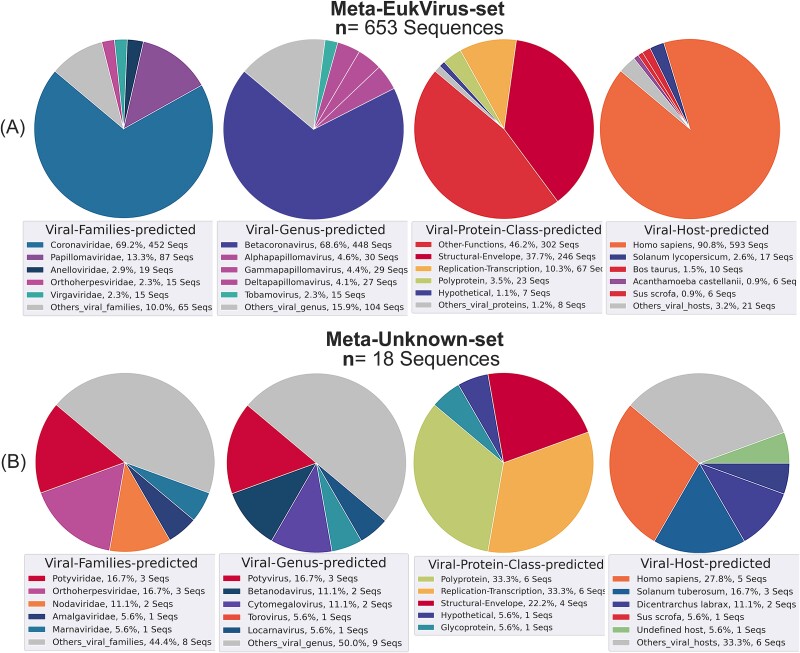
Classification results of VirDetect-AI on real metagenomic data across different levels (families, genera, protein types, and viral hosts). (A) Meta-EukVirus-set dataset. (B) Meta-Unknown-set dataset.

Moreover, sequences were identified as belonging to several viral families, such as *Coronaviridae* (69.2%), *Papillomaviridae* (13.3%), *Anelloviridae* (2.9%), and *Orthoherpesviridae* (2.3%) (Fig. 9A). *Betacoronavirus* emerged as the prevalent genus, covering 68.6% of the identified viral entities. Furthermore, *H. sapiens* was identified as the predominant host, covering 90.8% of the predicted sequences (Fig. 9A). Regarding viral protein functions, the three most frequently predicted categories were Other-Functions, Structural-Envelope, and Replication-Transcription. These results align with previously published data from the same metagenomic datasets [31]. Our analysis also identified less frequent but significant viral families such as *Paramyxoviridae* and *Flaviviridae* (Table S8), highlighting their clinical importance. Additionally, VirDetect-AI detected viruses infecting not only humans but also other vertebrates, invertebrates, and plants (Fig. 9A). This broad-spectrum identification underscores the robustness of our approach in accurately capturing the diversity of pathogenic viral entities across various host organisms.

The Meta-EukVirus-set was further evaluated using Blastp against a more recent NR viral database (as of 3 May 2023). Results indicate that 96.1% of total sequences (*n* = 703) were categorized as viral, with 3.9% yielding no hits (Fig. 8C). Among the classified viral entities (*n* = 676), 98.1% were categorized as eukaryotic viral proteins (Table S9). Most of these proteins belonged to families such as *Coronaviridae* (65.0%)*, Papillomaviridae* (13.3%), *Orthoherpesviridae* (3.8%), and *Iridoviridae* (3%).

The comparison of results between VirDetect-AI and Blastp revealed a clear similarity in their ability to classify eukaryotic viral proteins. Similar trends were observed in the percentage of families predicted. Notably, certain viral proteins showed <50% similarity to those in NR databases when analyzed using Blastp. This indicates that VirDetect-AI can identify novel viral proteins. In addition, VirDetect-AI produced fewer unclassified sequences in comparison to Blastp. This emphasizes the potential efficiency of VirDetect-AI over Blastp, which is a well-known tool for sequence comparison due to its accurate alignment. However, it is worth noting that Blastp requires significant processing resources [44].

The Meta-Unknown-set (*n* = 113), consisting of sequences that lacked homologs in the NR DB (September 2019) using Blastp, yielded interesting findings. VirDetect-AI categorized 29.2% of the entities as viral, 46.9% as NV, and 23.9% as unknown (Fig. 8A). Among the proteins categorized as viral (*n* = 33) (Table S10), 54.5% were assigned to eukaryotic viral classes, while 45.5% were classified as prokaryotic (Fig. 8B). This indicates the model’s capability to identify novel viral proteins, which may significantly differ from their reference sequences and thus may have been overlooked by Blastp. Diverse eukaryotic viral protein families were identified, such as *Potyviridae*, *Orthoherpesviridae*, and *Nodaviridae*, among others (Fig. 9B). Interestingly, in terms of the host, plants had the highest abundance (*n* = 6), followed by *H. sapiens* (*n* = 5), animals (*n* = 4), and insects (*n* = 2). These results are consistent with the host of other viral families reported in the reference article for these datasets [31].

Regarding the findings of the Meta-Human-set (*n* = 1280), which involved viral metagenomic sequences aligned to the human genome, VirDetect-AI determined that 10.1% were unclassified or unknown, and 69.8% were NV, suggesting that these are indeed accurate false negatives. Surprisingly, 16.5% (*n* = 211) of the sequences were classified by VirDetect-AI as eukaryotic viral (Fig. 8A), spanning 115 different classes or protein groups. Only five of these classes contained more than five sequences, indicating potential false positives. The sequences were categorized into protein groups of viral families such as *Orthoherpesviridae*, *Potyviridae*, *Retroviridae*, and *Secoviridae*, among others (Table S11). The reasons behind these erroneous assignments sequences were elucidated in the NCBI-EukVirusP-set analysis results (Evaluation on New Viral Sequences of NCBI Database Using VirDetect-AI, VirSorter2, and DeepVirFinder section).

The Meta-Human-set was subjected to additional testing using Blastp against the viral NR DB, resulting in similar findings. Specifically, 15.1% (*n* = 193) of the total sequences were classified as viral (Table S12). Consequently, false positives are likely to be present if sequences with homology to the host are not removed first. However, this approach may also result in authentic viral sequences being missed due to their integration into the host genome.

The classification results of the Meta-Bacteria-set (*n* = 2428) generated by VirDetect-AI, which included sequences of bifidobacteria species, were remarkable. A substantial proportion of sequences were categorized as NV (65.5%), suggesting a likely bacterial origin, whereas 6.4% remained unclassified, and 21.1% were categorized as prokaryotic-viral. Despite the fact that the prokaryotic-viral class seems abundant, it is important to consider potential overestimation because bifidoprophages comprise over 3% of the entire bifidobacterial pan-genome [34]. It is noteworthy that only 7% (*n* = 170) of the outcomes were assigned to 63 different eukaryotic viral classes, with only 10 having at least 5 sequences, indicating a relatively low incidence of false positives (Table S13).

The Meta-Bacteria-set was subjected to additional testing using Blastp against the NR viral database. The results yielded comparable results, ~2.2% of the sequences classified as eukaryotic viral proteins, indicating that Blastp provides similar identifications (Table S14). Consequently, VirDetect-AI generated only 4.8% of inaccurate eukaryotic identifications. These erroneous results exhibit low sequence query coverage, around 30%, suggesting that only a fragment of the sequence shows homology to viruses (Table S14). Interestingly, some of these sequences contain conserved domains that are prevalent across a wide range of proteins found in large bacterial, eukaryotic, and DNA viruses [38]. Furthermore, Blastp categorized 22.2% of the sequences as prokaryotic-viral, which further supports the precise identification of bifidophages.

Although VirDetect-AI was not specifically designed for predicting prokaryotic viral proteins, it demonstrates exceptional proficiency in their identification. Furthermore, it effectively classified most sequences as belonging to the negative class, which comprises protein sequences originating from bacteria.

While VirDetect-AI demonstrates strong classification performance, the speed of processing viral metagenomic sequences is equally critical in bioinformatics pipelines. Blastp, utilizing local alignments and substitution matrices, assesses sequence similarity with a computational cost of *O*(m^*^n), where *m* is the query sequence length and *n* is the database size. Hence, Blastp is slow with numerous sequences. We compare VirDetect-AI’s execution time, both with and without GPU acceleration, to Blastp on equivalent architectures (Table 4). On all datasets, VirDetect-AI outperforms Blastp v.2.11.0 on graphics processing unit (GPU) and central processing unit (CPU) systems, with analyses running 2120–4221 times faster with GPU acceleration and 14–33 times faster with CPU processing. These results underscore VirDetect-AI’s faster execution time across various datasets.

**Table 4:** Comparison of execution time of the experimental dataset.

**Dataset** ^**a**^	**Sequences per dataset**	**Execution Time (s)**		
		**VirDetect-AI**		**Blastp 2.11.0**
		**GPU**	**CPU**	**CPU**
Meta-EukVirus-set	703	170.4	35 762	656 862
Meta-Unknown-set	113	6.1	384.1	12 976
Meta-Human-set	1280	42.9	8862	181 088
Meta-Bacteria-set	2428	126.3	27 677	391 607
NCBI-EukVirusP-set	36 660	4339	Not applicable	Not applicable

^a^For all dataset experiments, the model VirDetect-AI ran on a machine with processors Intel(R) Core (TM) i9-12900K 12th Gen@3.2GHz and 128 GB of RAM memory and a GPU NVIDIA GeForce RTX 3080 with 12 GB of memory. Blastp runs on the same architecture.

## Conclusion

Current AI tools primarily focus on prokaryotic viral sequences, neglecting the classification of eukaryotic viral sequences crucial for public health [2, 7, 10, 11]. While some tools address eukaryotic viral contigs, they often provide binary classifications and lack comprehensive information, limiting their applicability to certain metagenomic samples. Our study addresses these shortcomings by providing VirDetect-AI, a deep learning tool that can be used in a variety of environments and is primarily intended to reliably categorize eukaryotic viral protein sequences.

VirDetect-AI exhibits robust performance throughout both the training and evaluation phases. During training, the model achieved exceptional accuracy with a rate of 0.99 and a loss of 0.02. Validation results further corroborate the model’s robustness, demonstrating an accuracy of 0.99 and a loss of 0.03. In the Test dataset, VirDetect-AI achieved excellent results across all performance metrics, including sensitivity, precision, F1-score, and MCC. Notably, 94.4% of the classes (*n* = 980) attained a sensitivity >0.9, while only 1.3% of the classes (*n* = 13) had sensitivities below 0.7. These findings highlight the model’s capability to accurately identify both viral and NV sequences in previously unseen data.

VirDetect-AI demonstrates high accuracy in identifying viral proteins across a diverse range of datasets, including both genuine viral metagenomic samples and novel NCBI viral proteins. It consistently achieves precise classification across 86 viral families and 521 genera and successfully identifies sequences from 43 novel viral families not included in the training database. Moreover, VirDetect-AI outperforms DeepVirFinder and VirSorter2 in analyzing novel NCBI viral sequences, and it exhibits superior execution speed compared to Blastp across metagenomic datasets. These attributes highlight VirDetect-AI’s efficiency and scalability for large-scale sequence analysis tasks.

However, VirDetect-AI has some limitations. The model occasionally produces false positives and false negatives, particularly in viral protein families with conserved global domains that are common in certain bacterial and eukaryotic sequences. Additionally, VirDetect-AI experiences a loss in sensitivity for some viral families, such as *Orthoherpesviridae*, *Nimaviridae*, *Mimiviridae*, *Narnaviridae*, *Potyviridae*, and *Retroviridae*. As discussed in the results, this decreased sensitivity may partly stem from the model’s exclusion of classes with fewer than 30 sequences, which can impair its ability to accurately identify rarer or less-represented viral proteins. Another contributing factor could be the high similarity between some of these viruses and the human genome, particularly for herpesviruses and retroviruses, which complicates the differentiation of viral proteins from human proteins and further impacts the model’s performance. The model’s effectiveness is also constrained by the requirement for contig lengths of at least 300 amino acids, which limits its ability to classify shorter sequences. Lastly, VirDetect-AI currently includes only a single class for prokaryotic viral sequences; further subdivision is needed to adequately cover the diverse viral families that infect bacteria.

**Key Points**

- VirDetect-AI is a deep learning tool that uses the power of convolutional neural network–residual neural networks to identify eukaryotic viral proteins in metagenomic data from diverse habitats and describes classes in detail.
- VirDetect-AI was designed for eukaryotic viral classes but has demonstrated the capability to identify prokaryotic viruses in a general manner.
- VirDetect-AI outperforms DeepVirFinder and VirSorter2 tools in analyzing novel NCBI viral sequences.
- VirDetect-AI significantly reduces processing time required to identify viral proteins compared to Blastp.

## Supplementary Material

Supplementary_Figures_VirDetect-AI_bbaf001

Supplementary_Tables_VirDetect-AI_v3_bbaf001

## Data Availability

The VirDetect-AI model, along with the program and implementation requirements, is available on GitHub at https://github.com/alyzart22/VirDetect-AI/. Additionally, results from novel NCBI viral sequences and from metagenomic datasets are included in this repository. The training, validation, and testing sequence data can be accessed on Zenodo at https://zenodo.org/doi/10.5281/zenodo.13328820. Additionally, this repository includes the data of novel NCBI viral sequences and metagenomic datasets.
