# Supplementary material for: VirDetect-AI: a residual and convolutional neural network–based metagenomic tool for eukaryotic viral protein identification
Source: Brief Bioinform. 2025 Jan 14;26(1):bbaf001. doi: 10.1093/bib/bbaf001 (PMC11729733; doi:10.1093/bib/bbaf001)
Supplement: Supplementary_Figures_VirDetect-AI_bbaf001 [file supplementary_figures_virdetect-ai_bbaf001.docx]

**
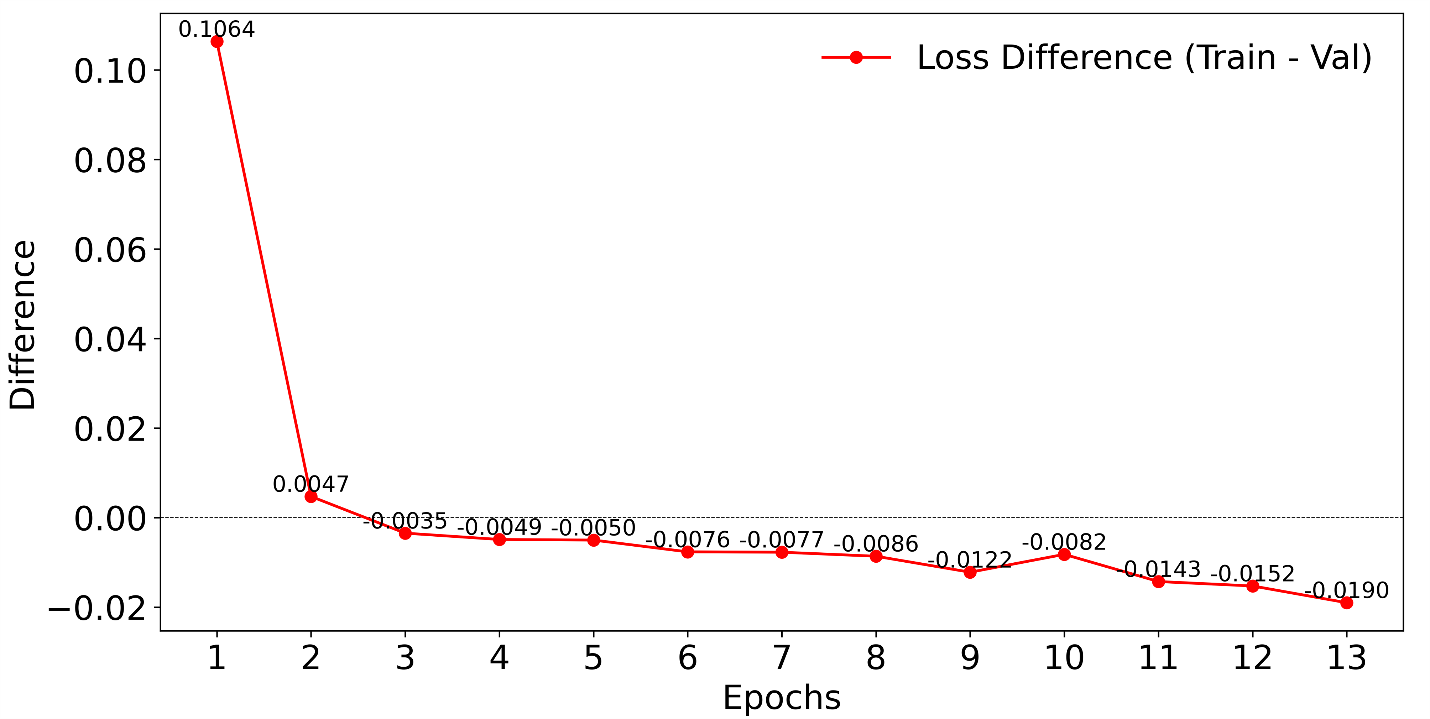
**

**Figure S1**. The Red line represents the difference between the loss metric in the training and validation data.


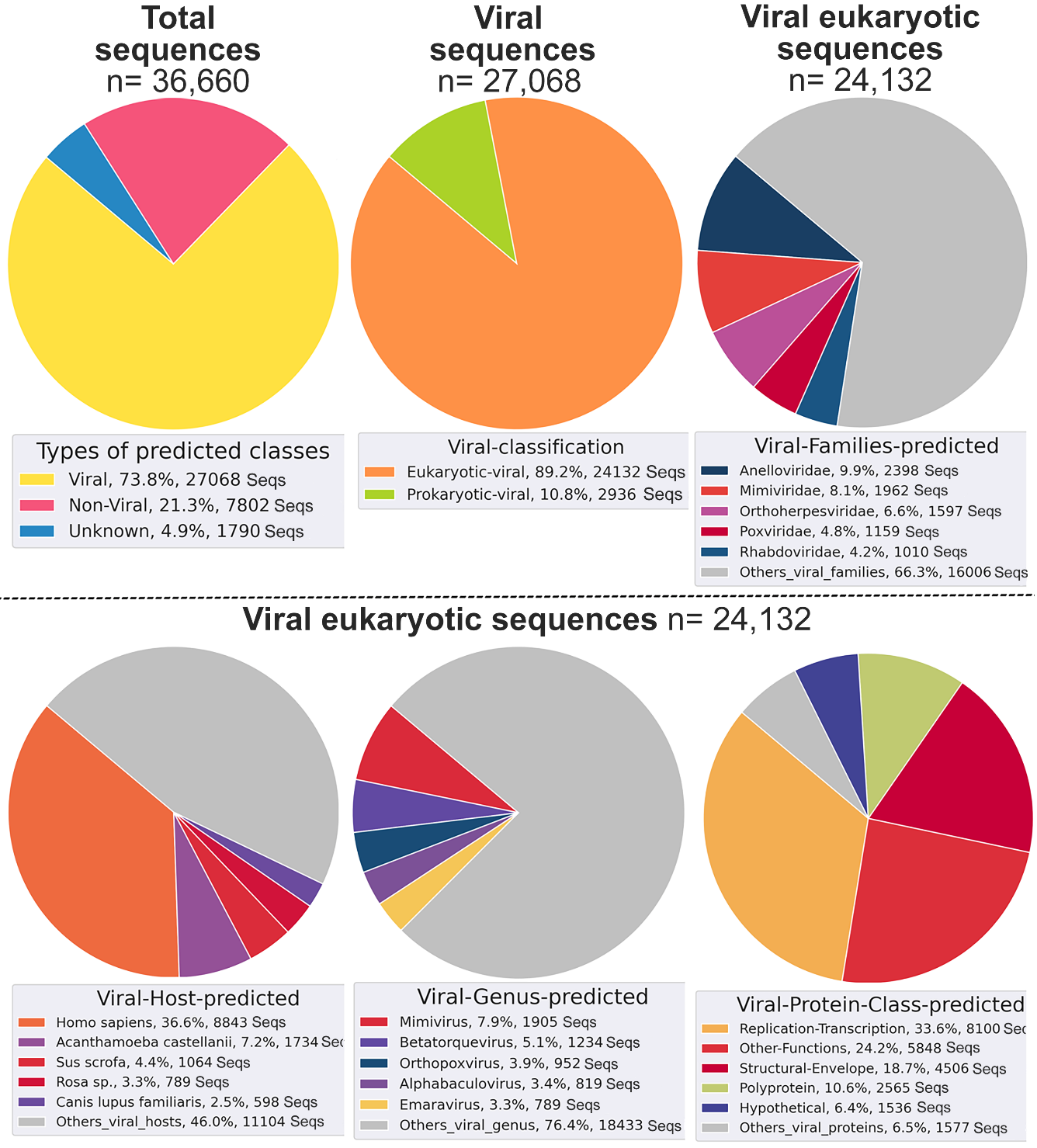


**Figure S2**. Classification results of VirDetect-AI on NCBI-EukVirusP-set.


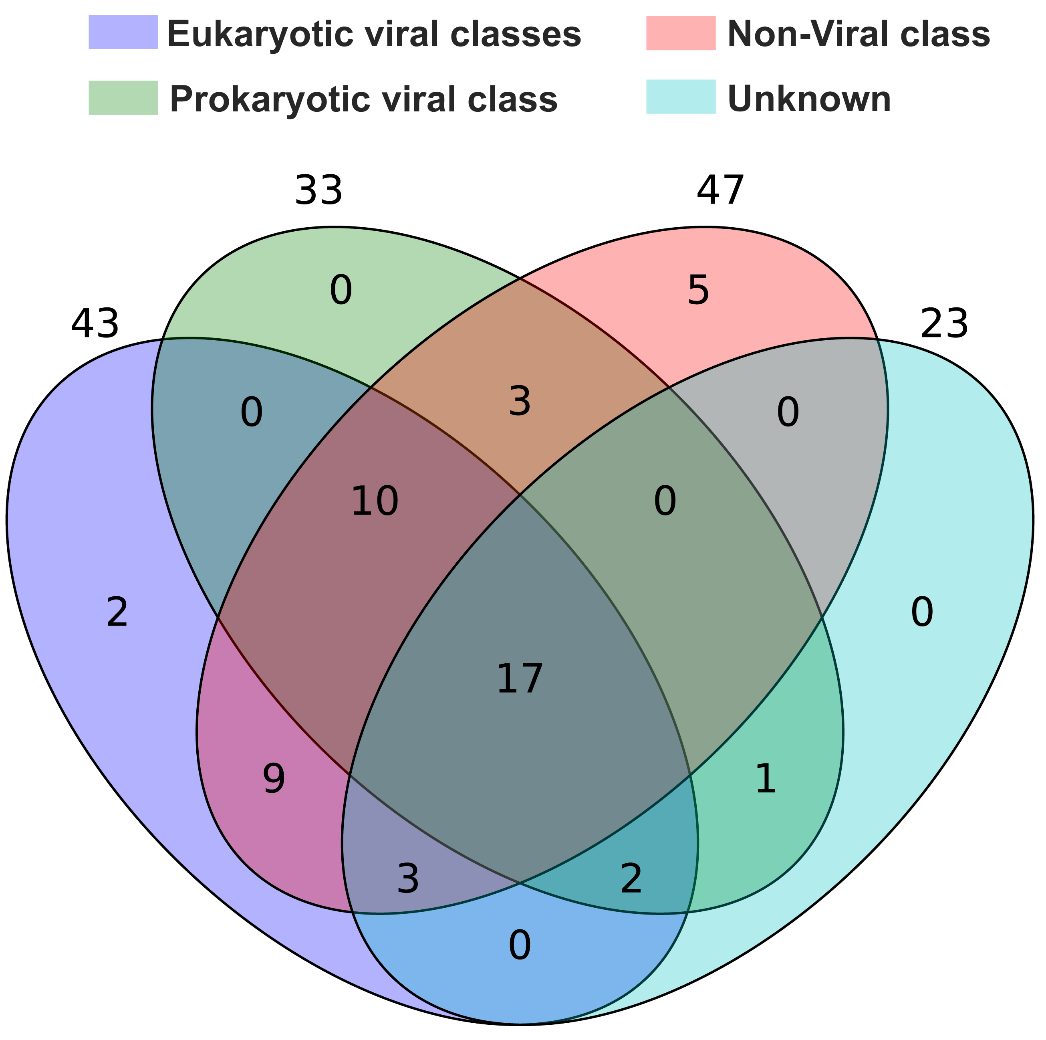
**Figure S3.** Venn diagram illustrating the classification outcomes of viral sequences from 52 newly identified families in the NCBI-EukVirusP-set, which were not included in the development of VirDetect-AI. Sequences were categorized into four classes: eukaryotic viral classes (purple), prokaryotic viral classes (green), non-viral classes (red), and unknown (cyan). The numbers in each section represent the count of families whose sequences were assigned to each category, with overlaps indicating families whose sequences were classified into multiple categories.
